# Case of Hydrophobia, Successfully Treated by the Scutellaria Lateriflora

**Published:** 1820-06

**Authors:** 


FOR THE LONDON MEDICAL AND PHYSICAL JOURNAL.

### *Case of Hydrophobia, successfully treated by the Scutellaria Lateriflora.* By Dr. Stillwell.

EARLY on Thursday morning, the 10th June, I was called upon by James Cann, who requested me to dress his right hand, which had just been bitten by a dog, which he believed was mad. Upon examination, I found the dog's teeth had penetrated deep into the muscular part of the thumb, between its metacarpal bone and that of the fore-finger, and that the skin was but little lacerated. From the situation and depth of the wound, I deemed extirpation inexpedient, and directed superficial bleedings; telling him at the same time, if the dog should prove to have been mad, he had nothing to fear, as a plant had been discovered, (showing him a drawing of the scullcap in the *Evening Post*,) which had never been known to fail in such cases, when properly administered. In the evening I saw him again, and then advised him to call on Jesse Williams, the son-in-law of the late Mr. Lewis, of West-Chester, and procure from him a quantity of scullcap. He did so, and obtained about three ounces of the dried herb, finely cut up, with directions to put a tea-spoonful and a half of it in a quart of warm water, and to drink half-a-pint of this infusion, morning and night, for two successive days; and on the third to omit it, and take a tea-spoonful of flowers of sulphur. In this manner, Williams directed the scullcap and sulphur to be alternately used for forty days; during which time, exercise was to be avoided, and an abstemious diet observed: he thought the wound required no other attention than simple dressing.

Mr. Cann strictly followed the above directions, and remained free from complaint till Thursday the 17th. About noon he was suddenly taken ill, and sent for me. I found him labouring under frightful spasms of the muscles of the face and neck: his face was drawn towards the right shoulder, his head convulsively shaken, he ground his teeth with violence, his eyes had a wild and terrific stare, and his whole aspect was appalling; but the spasms soon subsided, and he became perfectly calm. Upon enquiry, I found he was first attacked with a shivering, then a pricking or tingling sensation about the parts bitten, extending over the hand and running up the arm, accompanied with slight involuntary twitchings of the muscles of the hand and arm: to these succeeded a sense of tightness about the chest and throat; immediately after which followed the convulsive action of the muscles of the face and neck above described. I found his pulse and breathing regular and natural during the intervals; but, when the paroxysms were approaching, they became hurried and irregular, and continued so till the spasms had gone off; when he complained of slight pain in the right breast, together with a soreness and stiffness of the back part of the neck. Liquids he took without difficulty, nor did pouring water from one vessel to another in his presence produce any perceptible distress; neither did the sight of the surface of a polished mirror, or the waving of a white curtain, sensibly affect him. His paroxysms returned at irregular intervals of from five to ten minutes; their duration being from one to two minutes. His bowels being constipated, I gave him a scruple of calomel, and directed him to drink his tea (which, upon inspection, I found very weak,) as strong as it could be made; to take it warm, and in as large quantities as his stomach would bear, using it as his only drink.

18*th.*—Early in the morning, Dr. Robson saw him with me, and continued to see him afterwards. We learnt that some unauthorised person had taken ten or twelve ounces of blood from his arm the night before; that his cathartic had operated freely during the night; he had taken largely of his tea, and thought himself better: the spasms however still severe, but not quite so frequent. We directed him to continue his tea as yesterday.

19*th.*—This morning we found him cheerful: he had passed a tolerably good night; feels much better than yesterday, his spasms moderating considerably both in violence and frequency. He still continued his tea as before. In the afternoon a shower of rain fell, at sight of which, and the rippling of the water in the gutter, his spasms returned in quick succession, and with more violence than they had done at any other time during the day, and produced in him such sensations, that (to use his own expression) he could not bear to look at it, and was obliged to turn away.

20*th.*—We saw him about noon: he was not so well; his spasms rather more frequent and severe, leaving him with a disagreeable feeling in his head, and an acute pain in the back of his neck. Upon inquiring whether he still continued his tea, he replied, that, at Williams' direction, it was omitted for the purpose of taking a dose of sulphur; on which we immediately ordered his scullcap to be resumed, and not again to omit it, unless directed by us: he did so, and again found his spasms to subside.

21*st.*—He said he felt like a new man: his spasms had nearly left him; still continued in the use of his tea as before.

22*d.*—He had no spasms, nor did he complain of any thing but weakness. We directed him to continue in the use of the scullcap three or four weeks longer.

*July* 13*th.*—We saw him: he felt no uneasiness whatever, and has been free from complaint ever since we last visited him.

To enable the reader to form just conclusions respecting the character of the above case, we will state the result of our inquiries and observations concerning the rabid state of the animal which had inflicted the bite.

The dog was young and gentle, and had never shown marks of ill-temper until the day before he bit Cann, when he snapped at, and attempted to bite, a man, without provocation, who heretofore had been familiar with him. He was confined over night, but broke loose early next morning, the 10th, when

Cann, on his way to work, met him. The dog came trotting along, and Cann thinks would have gone on without noticing him, if he had not, when opposite, called him by name, and was in the act of patting his head, when the dog seized him by the hand, made two snaps, and passed on without looking up. A few yards further, he snapped at, and quarrelled with, three strange dogs; he next bit a neighbour's dog, with whom he was accustomed to play; and, as an apprentice of his master was attempting to tie him with a rope, he snapped at and tore off a part of his trowsers.

Behaviour like this, so opposite to his usual mildness, excited serious apprehension: he was immediately tied in a wood-house. While thus confined, he ate sparingly, but lapped water freely; he snapped at his master, was restless, howling violently, and gnawing furiously at the door of his prison. By the evening, when we saw him, he had gnawed a large hole through the door; in doing which, he had lacerated his mouth, and broken off several of his teeth against the nails of the batting. At this time, after many attempts, he lapped a little water, and then upset the vessel which contained it; refused food, and snapped at the approach of his master. His eyes were watery and dull, sometimes closed, then suddenly opened, when he snapped at imaginary objects. He now broke his rope; and, as no one dared to approach him to replace it, believing him mad, he was shot. Our next inquiry was after the dogs which had been bitten by this one; but we found they had all been destroyed, except the one last mentioned.

This dog was secured the same day he was bitten, and put in a cool, airy, and dry cellar; he was regularly fed, and continued well until the 6th of July. He then began to show symptoms of canine madness: the under jaw fell; his food dropped from his mouth, when he attempted to eat; he made many efforts to drink, frequently burying his nose in the water, but did not appear to swallow; he was obedient to his master's commands, was dull and moping, but would occasionally snap at imaginary objects, in the air or on the floor; his eyes were languid and watery, and considerable frothy saliva was discharged from his mouth. In the course of the next day (the 7th) he was much weaker, particularly in the hinder parts, producing slight staggering; his tongue was livid and brown: slimy fluid was observed to run out of his mouth. On the 8th, he would snap at his chain, or any object that touched him; was thirsty, and lapped water very frequently, without being able to swallow any; his tongue was darker, and his debility increased rapidly; he would not eat, and staggered very much when he attempted to walk.

*9th.*—The dog appeared much weaker; seldom got up, except by compulsion, and soon fell down again. He appeared blind in his right eye, his back much curved.

10*th.*—He was unable to stand; had spasmodic twitchings of all his muscles; would yet snap at any object that touched him. Towards evening he grew worse, and died sometime in the night.

The above statement of facts was drawn up for publication at the request of several respectable gentlemen, and is submitted without remark.

